# Postoperative Adverse Outcomes in Intellectually Disabled Surgical Patients: A Nationwide Population-Based Study

**DOI:** 10.1371/journal.pone.0026977

**Published:** 2011-10-27

**Authors:** Jui-An Lin, Chien-Chang Liao, Chuen-Chau Chang, Hang Chang, Ta-Liang Chen

**Affiliations:** 1 Department of Anesthesiology, Taipei Medical University Hospital, Taipei, Taiwan; 2 School of Medicine, College of Medicine, Taipei Medical University, Taipei, Taiwan; 3 Graduate Institute of Clinical Medicine, College of Medicine, Taipei Medical University, Taipei, Taiwan; 4 Department of Emergency Medicine, Shin Kong Memorial Hospital, Taipei, Taiwan; 5 Graduate Institute of Injury Prevention and Control, College of Public Health and Nutrition, Taipei Medical University, Taipei, Taiwan; 6 Taiwan Joint Commission on Hospital Accreditation, New Taipei City, Taiwan; University of Maryland, School of Medicine, United States of America

## Abstract

**Background:**

Intellectually disabled patients have various comorbidities, but their risks of adverse surgical outcomes have not been examined. This study assesses pre-existing comorbidities, adjusted risks of postoperative major morbidities and mortality in intellectually disabled surgical patients.

**Methods:**

A nationwide population-based study was conducted in patients who underwent inpatient major surgery in Taiwan between 2004 and 2007. Four controls for each patient were randomly selected from the National Health Insurance Research Database. Preoperative major comorbidities, postoperative major complications and 30-day in-hospital mortality were compared between patients with and without intellectual disability. Use of medical services also was analyzed. Adjusted odds ratios using multivariate logistic regression analyses with 95% confidence intervals were applied to verify intellectual disability's impact.

**Results:**

Controls were compared with 3983 surgical patients with intellectual disability. Risks for postoperative major complications were increased in patients with intellectual disability, including acute renal failure (odds ratio 3.81, 95% confidence interval 2.28 to 6.37), pneumonia (odds ratio 2.01, 1.61 to 2.49), postoperative bleeding (odds ratio 1.35, 1.09 to 1.68) and septicemia (odds ratio 2.43, 1.85 to 3.21) without significant differences in overall mortality. Disability severity was positively correlated with postoperative septicemia risk. Medical service use was also significantly higher in surgical patients with intellectual disability.

**Conclusion:**

Intellectual disability significantly increases the risk of overall major complications after major surgery. Our findings show a need for integrated and revised protocols for postoperative management to improve care for intellectually disabled surgical patients.

## Introduction

Intellectual disability (ID), defined as lower-than-normal intellectual function, is the most common developmental disorder [1]. It has a prevalence rate of 0.7–8.0% in various forms of severity [2] and is associated with a wide range of primary or secondary medical conditions complicating health management [2]. Earlier studies show severe ID as a negative predictor of life expectancy [3], and with more comorbidities compared with the general population [4]. Therefore intellectual disability's associated social services burden and related health care costs are high [5].

Patients with ID are susceptible to delayed diagnosis and adverse surgical outcomes due to associated anomalies, impaired communication and variable responses to pain and drugs [6]. A recent report found deaths of patients with ID are largely due to preventable causes and that these deaths result from poor medical practice [7].

A large-scale analysis of the global features of perioperative morbidity and mortality in surgical patients with ID is still lacking. Therefore we attempt to clarify whether ID is an independent risk factor for in-hospital major surgeries, and to validate the postoperative adverse outcomes in patients with ID.

## Methods

### Source of data and patient population

This study used reimbursement claims data from Taiwan's National Health Insurance Program, a universal insurance program that united 13 previous health insurance schemes starting from March 1995. More than 99% of the 22.6 million residents of Taiwan are enrolled in this system. The National Health Research Institutes established a National Health Insurance Research Database (NHIRD) that includes patient demographics and primary and secondary diagnoses of diseases, procedures, prescriptions and medical expenditures. It also records all reimbursements for inpatient and outpatient medical services. To protect personal privacy, the electronic database was decoded with patient identifications scrambled for further public access. The study was evaluated and approved by the NHIRD research committee.

We examined medical claims and identified 3983 surgical patients with preoperative diagnosis of ID from 2,010,412 persons who underwent inpatient major surgeries (defined as surgeries requiring general, epidural or spinal anesthesia, as well as hospitalization of more than one day) from 2004 to 2007 in Taiwan. For each surgical patient with ID, we randomly selected four non-ID subjects matched by sex, age and types of surgery from surgical patient populations as controls to increase statistical power.

### Measures

We recorded preoperative major coexisting medical conditions from medical claims for the 24-month preoperative period. These illnesses included acute myocardial infarction, acute renal failure, chronic obstructive pulmonary disease, congestive heart failure, diabetes mellitus, hypertension, peripheral vascular disease, stroke and conditions requiring renal dialysis. We considered 30-day postoperative mortality as the study's primary outcome. The 30-day postoperative complications including acute renal failure, deep wound infection, pneumonia, postoperative bleeding, septicemia, stroke and any complications were considered as secondary outcomes in the present study [8]. Following the *International Classification of Diseases, 9th Revision, Clinical Modification* (ICD-9-CM), we defined ID (ICD-9-CM 317–319) and other comorbidities and postoperative complications in this study, including hypertension (ICD-9-CM 401–405), chronic obstructive pulmonary disease (ICD-9-CM 490–496), diabetes mellitus (ICD-9-CM 250), acute myocardial infarction (ICD-9-CM 410), stroke (ICD-9-CM 430–438), congestive heart failure (ICD-9-CM 428), peripheral vascular disease (ICD-9-CM 443), acute renal failure (ICD-9-CM 584), deep wound infection (ICD-9-CM 958) , pneumonia (ICD-9-CM 480–486), postoperative bleeding (ICD-9-CM 998 and 999) and septicemia (ICD-9-CM 038, 998.0, and 778.52).

We calculated population density for each of Taiwan's 359 townships and city districts by dividing population by area (persons/km^2^). The first, second, third and fourth quartiles of population density were considered as areas of low, moderate, high and very high urbanization, respectively [9]. We noted whether the surgery took place in a teaching hospital or not and examined use of medical services in terms of length of hospitalization, use of intensive care unit and in-hospital medical expenditures. To validate whether the severity of ID was associated with postoperative complications, we classified ID into unspecified, mild to moderate, and severe to profound groups according to ICD-9-CM codes; the correlation of these groups with postoperative adverse outcomes was analyzed.

### Statistical analysis

Descriptive analyses concerning the distribution of demographic factors, coexisting medical conditions, intensive care unit stay and postoperative mortality and complication rates were compared between surgical patients with and without ID using chi-square tests. We used *t*-tests to compare the average of age, length of hospitalization and medical expenditure between surgical patients with and without ID.

Odds ratios (ORs) with 95% confidence intervals (CI) were analyzed for outcomes between patients with and without ID. Multivariate logistic regression with different models was used to adjust covariates such as surgery in a teaching hospital, low income, urbanization and preoperative coexisting medical conditions. In order to verify the impact of ID severity on 30-day postoperative mortality, we calculated adjusted ORs and 95% CI of postoperative complications associated with unspecified, mild to moderate, and severe to profound ID using multivariate logistic regression analysis. In order to deal with possible multiplicity issues, we applied a Bonferroni correction testing at a significance level of α (0.05)/k (number of tests) to lower the chance of a type 1 error. Data were analyzed with SAS software version 9.1 (SAS Institute Inc., Carey, NC, USA), with two-sided probability value of <0.05 considered statistically significant.

## Results

More ID patients were low-income (22.9% vs. 2.4%, p<0.001) and lived in less-urbanized areas (29.4% vs. 24.3%, p<0.001) compared with non-ID patients (Table S1). Compared with controls, ID patients were found to have higher prevalence of preoperative hypertension (12.9% vs. 9.1%, p<0.001), chronic obstructive pulmonary disease (26.8% vs. 15.3%, p<0.001), diabetes (9.5% vs. 5.4%, p<0.001), myocardial infarction (4.9% vs. 2.8%, p<0.001), stroke (9.3% vs. 2.3%, p<0.001), congestive heart failure (3.0% vs. 1.2%, p<0.001), peripheral vascular disease (1.0% vs. 0.5%, p = 0.001), dialysis (0.7% vs. 0.4%, p = 0.012) and acute renal failure (0.9% vs. 0.3%, p<0.001).

Surgical patients with ID had higher average length of hospitalization and use of intensive care, as well as higher rates of acute renal failure (1.0% vs. 0.3%, p<0.001) , pneumonia (4.5% vs. 1.7%, p<0.001), postoperative bleeding (3.6% vs. 2.4%, p<0.001), septicemia (2.9% vs. 0.1%, p<0.001), stroke (3.5% vs. 2.2%, p<0.001) and any complications (13.7% vs. 7.6%, p<0.001) (Table 1). In-hospital medical expenditures differed significantly between patients with and without ID when categorizing participants into a quartet with equal numbers of patients (p<0.001). However, no significant difference was found in postoperative 30-day mortality of patients with and without ID.

**Table 1:** Outcome characteristics of surgical patients with intellectual disability and controls.

	Preoperative intellectual disability				
	NoN = 15,932		YesN = 3,983		p value
Postoperative complications					
Acute renal failure	40	(0.3)	41	(1.0)	<0.001
Deep wound infection	141	(0.9)	43	(1.1)	0.251
Pneumonia	275	(1.7)	181	(4.5)	<0.001
Postoperative bleeding	384	(2.4)	142	(3.6)	<0.001
Septicemia	157	(1.0)	117	(2.9)	<0.001
Stroke	350	(2.2)	139	(3.5)	<0.001
Any of the above	1,207	(7.6)	544	(13.7)	<0.001
Length of stay, days					<0.001
1–5	9,710	(60.9)	1,978	(49.7)	
6–10	3,266	(20.5)	908	(22.8)	
11–15	1,126	(7.1)	324	(8.1)	
>15	1,830	(11.5)	773	(19.4)	
Mean±SD	8.2±12.7		11.9±20.0		<0.001
ICU stay, %	1,619	(10.2)	609	(15.3)	<0.001
In-hospital expenditure, USD*					
Low	721	(132.5)	775	(149.3)	<0.001
Medium	1,054	(88.1)	1,204	(160.0)	<0.001
High	1,637	(270.8)	2,046	(383.2)	<0.001
Very high	7,265	(7,446.2)	9,001	(6,997.2)	<0.001
Postoperative 30-day mortality	69	(0.4)	25	(0.6)	0.109

Parenthesis indicates the percentage of patients in each group.

ICU, intensive care unit.

*Mean (standard deviation).

After adjustment for confounding factors, surgical patients with ID had higher risks of postoperative acute renal failure (OR 3.81, 95% CI 2.28 to 6.37), pneumonia (OR 2.01, 1.61 to 2.49), bleeding (OR 1.35, 1.09 to 1.68), septicemia (OR 2.43, 1.85 to 3.21) and overall complications (OR 1.53, 1.35 to 1.73). Analyzed by multivariate logistic regression, postoperative 30-day mortality showed no significant difference between patients with or without ID after adjusting teaching hospital, low income, urbanization and coexisting disease (OR 1.52, 95% CI 0.91 to 2.54) (Table 2). The further analysis after Bonferroni correction on ID severity showed higher risk of postoperative septicemia in unspecified ID (OR 2.77, 95% CI 1.98 to 3.87), mild to moderate ID (OR 1.87, 1.29 to 2.71), and severe to profound ID (OR 4.12, 2.34 to 7.28) (Table 3). The corresponding ORs for overall postoperative complications associated with unspecified, mild to moderate, and severe to profound ID were 1.72 (95% CI 1.46 to 2.03), 1.26 (1.07 to 1.49) and 2.40 (1.74 to 3.31), respectively.

**Table 2:** Risk of postoperative 30-day mortality and complications associated with intellectual disability in multiple logistic regression models*.

	OR	(95% CI)
Postoperative 30-day complications		
Acute renal failure	3.81	(2.28–6.37)
Deep wound infection	1.32	(0.91–1.90)
Pneumonia	2.01	(1.61–2.49)
Postoperative bleeding	1.35	(1.09–1.68)
Septicemia	2.43	(1.85–3.21)
Stroke	1.18	(0.91–1.53)
Any of the above	1.53	(1.35–1.73)
Postoperative 30-day mortality	1.52	(0.91–2.54)

*Adjusted for age, sex, types of surgery, teaching hospital, low income, urbanization, and coexisting diseases.

OR, odds ratio; CI. Confidence interval.

**Table 3:** Risk of postoperative 30-day mortality and complications by severity of intellectual disability in multiple logistic regression models*.

	Severity of intellectual disability							
	No disabilityN = 15,932		UnspecifiedN = 1,660		Mild to moderateN = 2,023		Severe to profoundN = 300	
Postoperative 30-day complications	OR	(95% CI)	OR	(95% CI)	OR	(95% CI)	OR	(95% CI)
Acute renal failure	1.00	(reference)	4.67	(2.61–8.36)†	3.04	(1.52–6.07)†	2.67	(0.71–10.0)
Deep wound infection	1.00	(reference)	1.56	(0.96–2.52)	1.18	(0.73–1.92)	0.86	(0.21–3.58)
Pneumonia	1.00	(reference)	2.51	(1.92–3.26)†	1.36	(1.00–1.84)	3.75	(2.36–5.97)†
Postoperative bleeding	1.00	(reference)	1.59	(1.20–2.10)†	1.13	(0.84–1.51)	1.65	(0.92–2.95)
Septicemia	1.00	(reference)	2.77	(1.98–3.87)†	1.87	(1.29–2.71)†	4.12	(2.34–7.28)†
Stroke	1.00	(reference)	1.30	(0.93–1.80)	1.09	(0.77–1.53)	0.98	(0.44–2.22)
Any of the above	1.00	(reference)	1.72	(1.46–2.03)†	1.26	(1.07–1.49)†	2.40	(1.74–3.31)†
Postoperative 30-day mortality	1.00	(reference)	1.99	(1.08–3.64)	0.91	(0.40–2.06)	2.53	(0.74–8.65)

*Adjusted for age, sex, types of surgery, teaching hospital, low income, urbanization, and coexisting diseases.

OR, odds ratio; CI, confidence interval.

† Statistically significant after Bonferroni correction (p<0.0125) (α = 0.05/4 tests = 0.0125).

## Discussion

This large-scale, population-based and cross-sectional study shows that compared with controls without intellectual disability, surgical patients with ID had significantly higher incidence of preoperative comorbidities and postoperative complications while consuming more medical resources. After adjustment for confounding variables, risks of overall postoperative 30-day complications increased in surgical patients with ID and highly correlated with ID severity, especially in septicemia.

The prevalence of severe ID in low-income countries is at least double that in high-income countries [10]. Malnutrition, iodine deficiency, birth trauma and lead poisoning are all suspected factors in this difference in prevalence [5]. A previous study found 61.3% of patients with ID belong to rural populations with low socioeconomic status [6]. Our result also showed that surgical patients with ID had lower incomes and lived in less-urbanized areas compared with the control group.

Respiratory diseases, followed by cardiovascular complications, are the leading causes of death in patients with ID [11], and pneumonia is the most common lethal illness [3]. Earlier reports showed ID is a risk factor of postoperative pulmonary complications [12], [13], which is compatible with our data that surgical patients with ID were at higher risk of developing pneumonia than patients without ID. Risk of aspiration pneumonia in patients with ID is substantially high due to multiple factors, including ID-associated gastroesophageal reflux [14], dysfunctional pharyngoesophageal sphincter [15], and anticonvulsants and/or tranquilizers that may cause hypersalivation when prescribed for ID-accompanying seizures [16]. Drugs for postoperative pain or agitation may also depress pharyngeal reflexes. In addition, significant physical impairment in ID patients further accentuates the risk of aspiration after surgery [17]. These studies confirm our results regarding intellectually disabled surgical patients' elevated risk of developing postoperative pneumonia.

Severe dental caries and periodontal disease were common handicaps associated with ID [18]. Poor oral hygiene is anticipated and leads to a higher rate of bacteremia after dental treatment in patients with ID than in others [19]; it is also reported as a risk factor for infective endocarditis [20]. As the most frequent genetic cause of ID [21], Down syndrome has been associated with inherent immunodeficiency, and it may precipitate sepsis progression and increase mortality risk in children with sepsis [22]. Our results showed the severity of ID positively correlated with risk of septicemia following surgery, suggesting that poorer oral hygiene or immunity possibly correlates with disability severity.

Renal dysfunction has been reported in many ID-related syndromes, such as Williams-Beuren syndrome [23], Bardet-Biedl syndrome [24], oculocerebrorenal syndrome of Lowe [25] and Menkes disease [26]. Polycystic kidney can manifest as a ID-accompanying disorder, and all such patients progress to chronic renal failure early in their lives [27]. Our results showed the relative risk of acute renal failure was highest among major postoperative complications, which indicates that kidney function is most easily impaired by perioperative health care management in surgical patients with ID.

Several coexisting clotting defects have been reported in patients with ID, including factor V [28], factor VII (alone [29] or in combination with factor X [30]), and factor XI (alone [31] or in combination with factor XII [32]). Complicated bleeding diathesis (decreased factor XI, XII, von Willebrand's factor and platelet dysfunction) in some ID patients might be further exacerbated by physiological alterations in specific conditions (such as pregnancy) and result in postoperative bleeding [33]. The fragile X-ID syndrome, as one of the major causes of genetically determined ID, has a closely linked fragile mutated site with haemophilia B loci [34]. Bleeding resulting from therapy-related side effects is also a critical issue in these cases. Lorenzo's oil, as a choice of dietary management for ID patients with adrenoleukodystrophy, may cause thrombocytopenia [35]. Epilepsy was reported to be the most prevalent illness in patients with ID in Taiwan [36]; therefore valproate-associated coagulopathies should not be overlooked, even during short-term epileptic treatment [37], as risk of postoperative bleeding is increased.

Respiratory complications were the leading cause of death in patients with ID [11]. The incidence of postoperative pulmonary complications could be reduced significantly by routinely nursing patients in humidity tents for 24–48 h and by commencing vigorous chest physiotherapy immediately after surgery in patients with ID [14]. Moreover, perioperative antibiotic coverage and aggressive pulmonary hygiene are also crucial to prevent postoperative pulmonary complications in ID patients with high susceptibility, including congenital central alveolar hypoventilation syndrome (Ondine's curse) [38], Langer-Giedion syndrome [39], mucopolysaccharide storage disorders [40] and cri du chat syndrome [41]. ID patients with congenital insensitivity to pain with anhidrosis should be managed as “full stomach” patients to prevent perioperative regurgitation and aspiration, because their gastric emptying is delayed due to autonomic nervous system dysfunction [42].

This study is limited to retrospective reimbursement claims without individual detailed biochemical data. A definite relationship between preoperative comorbidities and postoperative complications for each surgical patient with or without ID could not be verified. Information about physical activity, degree of family support and loading of caretakers was also unavailable in this study. Another limitation is that we investigated the global adverse outcome focusing on all ID patients receiving in-hospital surgeries. However, patients with definite diagnosis, such as Down syndrome, were not specified in our study due to patient collection in our study design as mental retardation (ICD-9-CM 317–319).

In conclusion, our results are the first to validate through a population-based study that risks for postoperative major complications were increased in patients with intellectual disability and correlated with ID severity. These findings confirmed the higher risks in surgical patients with ID for in-hospital major surgery and a corresponding need to provide integrated health care for both prevention and postoperative management. Efforts should be made to reallocate adequate resources for these purposes [5], and strategies are needed to reduce postoperative adverse outcomes in this population.

## Supporting Information

Table S1 Demographic factors and preoperative medical conditions in surgical patients with and without intellectual disability^*^.
(DOCX)
