# Supplementary material for: Postoperative Adverse Outcomes in Intellectually Disabled Surgical Patients: A Nationwide Population-Based Study
Source: PLoS One. 2011 Oct 27;6(10):e26977. doi: 10.1371/journal.pone.0026977 (PMC3203162; doi:10.1371/journal.pone.0026977)
Supplement: Table S1 — Demographic factors and preoperative medical conditions in surgical patients with and without intellectual disability*. (DOCX) [file pone.0026977.s001.docx]

Supporting Information Legends

| Table S1 Demographic factors and preoperative medical conditions in surgical patients with and without intellectual disability^*^ | | | | | |
| --- | --- | --- | --- | --- | --- |
|  | Preoperative intellectual disability | | | |  |
|  | No (N=15,932) | | Yes (N=3,983) | | p value |
| Sex | n | (%) | n | (%) | 1.000 |
| Female | 6,016 | (37.8) | 1,504 | (37.8) |  |
| Male | 9,916 | (62.2) | 2,479 | (62.2) |  |
| Age, years |  |  |  |  | 1.000 |
| 0-9 | 2,632 | (16.5) | 658 | (16.5) |  |
| 10-19 | 3,356 | (21.1) | 839 | (21.1) |  |
| 20-29 | 3,692 | (23.2) | 923 | (23.2) |  |
| 30-39 | 1,976 | (12.4) | 494 | (12.4) |  |
| 40-49 | 2,104 | (13.2) | 526 | (13.2) |  |
| 50-59 | 1,316 | (8.3) | 329 | (8.3) |  |
| ≥60 | 856 | (5.4) | 214 | (5.4) |  |
| Mean±SD | 28.5±18.4 | | 28.4±17.8 | | 0.460 |
| Types of surgery | n | ( %) | n | (%) | 1.000 |
| Skin | 952 | (6.0) | 238 | (6.0) |  |
| Breast | 176 | (1.1) | 44 | (1.1) |  |
| Musculoskeletal | 5,704 | (35.8) | 1,426 | (35.8) |  |
| Respiratory | 824 | (5.2) | 206 | (5.2) |  |
| Cardiovascular | 320 | (2.0) | 80 | (2.0) |  |
| Digestive | 3,304 | (20.7) | 826 | (20.7) |  |
| Kidney, ureter, bladder | 692 | (4.3) | 173 | (4.3) |  |
| Delivery, CS, abortion | 368 | (2.3) | 92 | (2.3) |  |
| Neurosurgery | 1,176 | (7.4) | 294 | (7.4) |  |
| Eye | 684 | (4.3) | 171 | (4.3) |  |
| Others | 1,732 | (10.9) | 433 | (10.9) |  |
| Operation in teaching hospital |  |  |  |  | 0.562 |
| No | 1,575 | (9.9) | 406 | (10.2) |  |
| Yes | 14,357 | (90.1) | 3,577 | (89.8) |  |
| Low income |  |  |  |  | <0.001 |
| No | 15,551 | (97.6) | 3,071 | (77.1) |  |
| Yes | 381 | (2.4) | 912 | (22.9) |  |
| Urbanization |  |  |  |  | <0.001 |
| Low | 3,867 | (24.3) | 1,171 | (29.4) |  |
| Moderate | 3,986 | (25.0) | 1,123 | (28.2) |  |
| High | 3,942 | (24.7) | 855 | (21.5) |  |
| Very high | 4,137 | (26.0) | 834 | (20.9) |  |
| Coexisting medical conditions |  |  |  |  |  |
| Hypertension | 1,442 | (9.1) | 514 | (12.9) | <0.001 |
| COPD | 2,430 | (15.3) | 1,068 | (26.8) | <0.001 |
| Diabetes mellitus | 856 | (5.4) | 380 | (9.5) | <0.001 |
| Myocardial infraction | 448 | (2.8) | 193 | (4.9) | <0.001 |
| Stroke | 373 | (2.3) | 372 | (9.3) | <0.001 |
| Congestive heart failure | 191 | (1.2) | 118 | (3.0) | <0.001 |
| Peripheral vascular disease | 80 | (0.5) | 39 | (1.0) | 0.001 |
| Renal dialysis | 64 | (0.4) | 28 | (0.7) | 0.012 |
| Acute renal failure | 44 | (0.3) | 34 | (0.9) | <0.001 |
| ^*^Matched with sex, age, and types of surgery.  CS, Caesarean section; COPD, Chronic obstructive pulmonary disease | | | | | |
